# Role of Exercise Training on Autonomic Changes and Inflammatory Profile Induced by Myocardial Infarction

**DOI:** 10.1155/2014/702473

**Published:** 2014-06-18

**Authors:** Bruno Rodrigues, Fabio S. Lira, Fernanda M. Consolim-Colombo, Juraci A. Rocha, Erico C. Caperuto, Kátia De Angelis, Maria-Cláudia Irigoyen

**Affiliations:** ^1^Human Movement Laboratory, Sao Judas Tadeu University (USJT), Avenida Taquari 546, 03166-000 São Paulo, SP, Brazil; ^2^Immunometabolism Research Group, Department of Physical Education, Universidade Estadual Paulista (UNESP), Rua Roberto Simonsen 305, 19060-900 Presidente Prudente, SP, Brazil; ^3^Hypertension Unit, Heart Institute (InCor), Medical School of University of São Paulo, Avenida Dr. Enéas de Carvalho Aguiar 44, 05403-900 São Paulo, SP, Brazil; ^4^Translational Physiology Laboratory, Universidade Nove de Julho (UNINOVE), Rua Vergueiro 235/249, Liberdade, 01504-001 São Paulo, SP, Brazil

## Abstract

The cardiovascular autonomic imbalance in patients after myocardial infarction (MI) provides a significant increase in mortality rate, and seems to precede metabolic, hormonal, and immunological changes. Moreover, the reduction in the parasympathetic function has been associated with inflammatory response in different pathological conditions. Over the years, most of the studies have indicated the exercise training (ET) as an important nonpharmacological tool in the management of autonomic dysfunction and reduction in inflammatory profile after a myocardial infarction. In this work, we reviewed the effects of ET on autonomic imbalance after MI, and its consequences, particularly, in the post-MI inflammatory profile. Clinical and experimental evidence regarding relationship between alterations in autonomic regulation and local or systemic inflammation response after MI were also discussed.

## 1. Introduction

Since the 50s, when cardiovascular diseases (CVDs) exceeded 50% as a cause of mortality worldwide, a detailed search for better understanding of the risk factors was initiated with the Framingham study. Cigarette smoking, hypertension, hypercholesterolemia, diabetes mellitus, physical inactivity, and obesity were identified as the main threats [1] and prevention strategies were initiated.

Despite all technological and scientific advances in the management and prevention of CVD, it remains the leading cause of morbidity and mortality in developed countries and it is fast becoming a major health challenge in developing countries, contributing significantly to high costs in public health [2]. Coronary artery disease (CAD) has a broad spectrum of clinical manifestations, in particular acute coronary syndromes (ACS), that is, unstable angina and acute myocardial infarction (MI). MI is estimated to occur in the US every 44 seconds and about 49% of CVD deaths in the country are attributed to cardiac ischemic events [2].

Both during and after MI, neurohumoral changes occur in order to minimize the consequences of reduced ventricular function, which is caused by the obstruction of blood flow in the left ventricle (LV) of patients who had experienced an ischemic event. On the other hand, chronically, autonomic imbalance is a key element in the pathophysiology of heart failure (HF) after a MI [3–6]. Thus, strategies in order to detect, prevent, and attenuate the cardiovascular autonomic dysfunction, particularly associated with reduced vagal activity, have been seen as important interventions in the management of the changes triggered by MI.

The metabolic, cardiovascular, autonomic, and anti-inflammatory benefits of a physically active life style have led many researchers to suggest exercise training (ET) as an important nonpharmacological tool in the prevention and treatment of CVD [7–11]. The effectiveness of ET as a great tool in the treatment of patients with established CAD (either with or without MI) has been widely reported in the literature [7, 12–16]. In this sense, the purpose of this paper was to review the effects of ET on autonomic imbalance and inflammatory profile after MI. Clinical and experimental evidence, or lack of them, regarding relationship between alterations on cardiac autonomic regulation and local or systemic inflammation response after MI will be discussed.

## 2. Myocardial Infarction and Autonomic Dysfunction

**Figure 1:**
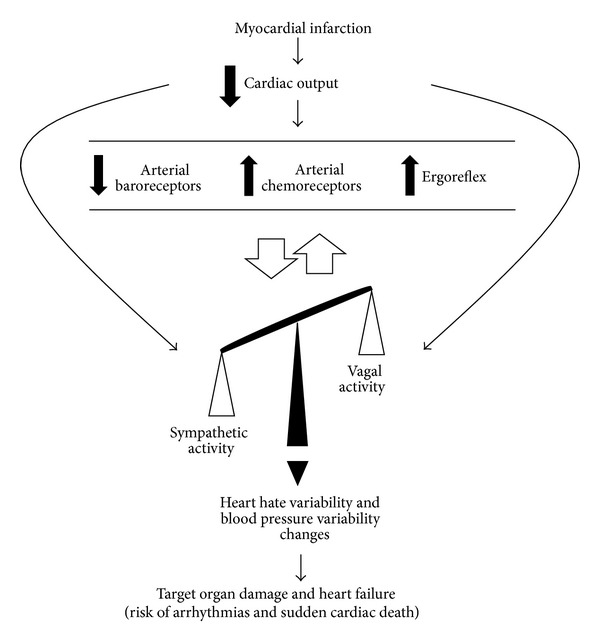
Cardiovascular reflexes impairment and cardiac autonomic nervous system imbalance after myocardial infarction.

As illustrated in Figure 1, after MI, the reduction in cardiac output is associated with an imbalance of the autonomic nervous system in favor of increased sympathetic activity and reduced vagal activity and is usually accompanied by abnormalities in the cardiorespiratory reflex control, that is, impairment of baroreflex sensitivity and function, and increased activation of ergoreflex and chemoreflex [3, 5, 17, 18]. The sympathoinhibition by the arterial baroreflex is significantly suppressed, whereas sympathoexcitatory reflexes, including the cardiac sympathetic afferent reflex, arterial chemoreceptor, and cardiopulmonary reflexes, are augmented [19]. Thus, the change in cardiovascular reflexes leads to a generalized activation of the sympathetic nervous system after MI in order to change the heart and peripheral hemodynamics. These changes are initially necessary; however, chronically, they are associated with reduced heart rate variability and increased blood pressure variability, which contributes with target organ damage, heart failure development, risk of arrhythmias, and sudden cardiac death [20].

In this sense, the reflex control of circulation commanded by arterial pressure receptors has been recognized as an important predictor of cardiovascular risk after cardiac event. The ATRAMI study (autonomic tone and reflexes after myocardial infarction) has provided clinical evidence of the prognostic value of baroreflex sensitivity and heart rate variability (HRV) in the mortality rate after MI, regardless of ejection fraction and ventricular arrhythmias [3]. Furthermore, in a study undertaken by Kleiger et al. [21], at the Multicenter Post Myocardial Infarction Program, it was found that the individuals who had lower HRV displayed a higher relative risk of mortality (five times) as compared with those who had higher HRV.

Additionally, experimental data have also pointed to a loss of autonomic function in animals after coronary artery occlusion. Indeed, our group has consistently demonstrated that after chronic MI the animals displayed baroreflex dysfunction and reduction in total HRV and in parasympathetic modulation, as well as increased sympathetic modulation in relation to the noninfarcted animals [19, 22–28]. Furthermore, the changes of hormonal/signaling factors levels at specific sites (angiotensin II, nitric oxide, reactive oxygen species, arginine vasopressin, endothelin-1, atrial natriuretic peptide, prostaglandins, and aldosterone), as well as the increased concentration of proinflammatory cytokines in the central nervous system, may be potential candidate mechanisms underlying the increased sympathetic outflow [29–33].

The clinical prognosis of increased sympathetic activity after myocardial ischemia and HF is now well established [34], and, as such, beta adrenergic blockade has become a standard element in the therapy of these patients [35]. In contrast, the reduction in parasympathetic tonus, although already demonstrated more than 40 years [36], has received less attention. In this sense, more recently, experimental studies have demonstrated that vagal stimulation promoted an antifibrillatory effect and reduced mortality rate in animal models of HF [37, 38]. On the other hand, our group has recently tested the effectiveness of treatment with pyridostigmine bromide, an acetylcholinesterase inhibitor, for 7 days (0.14 mg/mL/day), on autonomic function in rats after MI. The MI treated group improved vagal tonus and decreased sympathetic tonus and MI area in relation to the MI placebo group [39].

Thus, interventions to detect, prevent, or attenuate cardiovascular autonomic dysfunction (particularly reduced vagal activity) have been welcomed as new and important strategies in the management of the MI-induced changes.

## 3. Autonomic Dysfunction and Inflammatory Response 

Inflammatory processes combined with cytokine release are important steps in response to tissue injury and play an active role in cardiac remodeling and function after MI. Inflammatory cytokines, such as tumor necrosis factor-alpha (TNF-*α*), interleukin-1*β* (IL-1*β*), interleukin-6 (IL-6), and interleukin-10 (IL-10), are released after acute ischemic injury and may regulate survival or apoptosis of myocytes, as well as triggering additional inflammatory cell response [40]. In this sense, Ammirati et al. [41] have observed 109 patients during an acute MI and found that those with additional elevations of IL-6 and IL-10 displayed a poor prognosis when compared to individuals with lower levels of these cytokines. Thus, the authors suggested that the degree of acute inflammation after ischemia may indicate a poor prognosis in MI patients.

Chronically, cytokines may mediate cardiac remodeling and repair, enabling the formation of collagen and matrix metalloproteinase, associated with integrin regulation, angiogenesis, and mobilization of progenitor cells [40]. After the initial increase of proinflammatory cytokines in the infarcted area, their levels usually decline toward baseline values one week after MI [42]. However, according to Ono et al. [42], if the size of MI is large, or if there are other stress factors in course, the gene expression of cytokines may remain significantly elevated in noninfarcted region following 20 weeks of MI. Furthermore, these researchers showed that the cytokines levels, such as IL-1*β*, are associated with ventricular diastolic diameter increase and collagen deposition in the infarcted area after 8 and 20 weeks of MI. Thus, acutely and/or chronically, the release of proinflammatory cytokines adversely affects the LV function, exerts a negative inotropic effect [43], induces abnormalities in cardiac metabolism, and promotes myocardial remodeling, leading to HF [44, 45]. Additionally, activation of the immune system promotes the development of endothelial dysfunction and skeletal muscle apoptosis in HF [46, 47].

The origin of immune activation after ischemia has been dealt with by various research studies but remains unclear. There are at least five hypotheses addressing the underlying mechanism of inflammatory response [48]: (1) the failure of the myocardium* per se* would be the main source of cytokine production [49]; (2) the circulatory decompensation would lead to increased intestinal translocation of bacterial endotoxin (lipopolysaccharide) to the systemic circulation, which in turn would activate circulating immune cells [50]; (3) the main source of proinflammatory mediators would be the body tissues exposed to hypoxia [51, 52]; (4) immune activation would be a consequence of increased [53] sympathetic stimulation; and (5) reduction in parasympathetic participation would work as the primary mediator of the inflammatory response activation [48].

Studies by Kevin J. Tracey group [54–62] have lent strength to the hypothesis of a direct relation between parasympathetic activation and immune system response. This group initially argued that the activation of the vagus nerve (electrically or by cholinergic agonists) would reduce inflammatory response in experimental models of sepsis, and subsequently they postulated the theory of “inflammatory reflex,” linking aspects related to neuroimmunomodulation. The reflex arc, mediated by the nervous system, is composed by efferent via, integrative areas of central nervous system, and efferent via. Briefly, Kevin J. Tracey's group observed that inflammatory mediators (cytokines) produced in peripheral tissues may warn the central nervous system by a direct central action or by afferent stimulation of the vagus nerve. In this model, the integration of signals takes place in the central areas, triggering the activation of the parasympathetic efferent pathway, mediated mainly by the vagus nerve, whose neurotransmitter is acetylcholine. The cholinergic pathway innervates various components of the immune system (reticuloendothelial system), such as lymph nodes, liver, heart, spleen, and gastrointestinal tract. The activation of the vagus nerve leads to reduced production of cytokines, which in turn decreases the inflammatory response in models of septic and aseptic inflammation [55].

According to the “inflammatory reflex” theory, the vagus nerve seems to be the most important element in the efferent arm. In this sense, Blalock [63] has suggested that the immune system linked in such way to the central nervous system works as a “sixth sense,” being able to detect microbial invasion and other inflammatory substances and to retransmit this information to the brain, triggering responses that would interfere in the initial process. In fact, studies have viewed the parasympathetic hyperactivity, brought about by either drugs or direct vagal stimulation, as a mechanism that reduces the release of cytokines and reactive oxygen species during an inflammatory process [56, 64].

**Figure 2:**
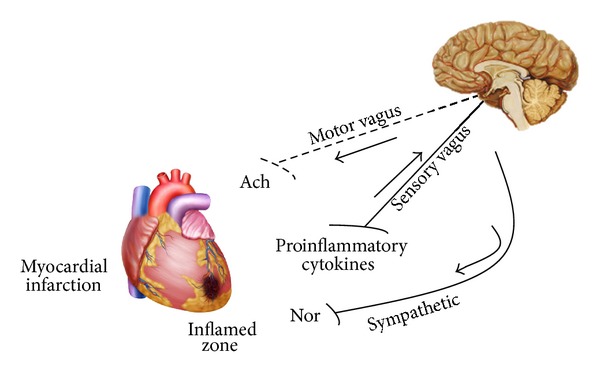
Blunted “inflammatory reflex” by vagal efferent function reduction after myocardial infarction (adapted from Tracey [55]). Ach: acetylcholine; Nor: noradrenaline.

In this context, Borovikova et al. [65] have also demonstrated that injection of endotoxin (lipopolysaccharide) in animals that underwent vagus nerve stimulation resulted in reduced systemic release of inflammatory cytokines and macrophages, without affecting the release of interleukin-10 (IL-10). However, the vagus nerve transection abolished this protection. Accordingly, in humans, significantly reduced HRV is associated with elevated levels of inflammatory cytokines (IL-6) and C-reactive protein (CRP) [66]. Lanza et al. [67] have shown that serum CRP levels were significantly associated with reduced HRV in patients with unstable angina. This association has also been observed in healthy individuals and patients with stable coronary artery disease and HF [68]. Thus, in accordance with “inflammatory reflex” theory, inflammatory products produced in ischemic ventricle activate afferent signals that are relayed to the nucleus tractus solitaries, and subsequent activation of vagus efferent activity would inhibit cytokine synthesis through the cholinergic anti-inflammatory pathway (Figure 2). However, as previously mentioned there is an imbalance in favor of increased sympathetic activity and reduced vagal activity after MI. Thus, it is possible to suggest that cardiovascular autonomic imbalance after ischemic event may blunt “inflammatory reflex” by reducing cholinergic anti-inflammatory pathway. However, despite the fact that some suggestions are pointed out on this direction in the literature [66–68], there is no strong evidence to support a cause-effect relationship.

In this sense, preventing and/or attenuating autonomic dysfunction triggered by MI would result in a less intense inflammatory response and, as such, cardiac and peripheral structure and function would be preserved.

## 4. Aerobic Exercise Training as Therapy

In recent years, our laboratory has investigated the changes induced by MI alone [25, 27] or associated with different risk factors, such as hypertension [69, 70], ovarian hormone deprivation [23], sinoaortic denervation [24], and diabetes [26–28, 71–73]. These studies have also tested different therapeutic approaches in the management of MI-induced changes. Among these therapeutic approaches, some deserve greater emphasis: therapy involving stem cells and mesenchymal bone marrow both* in situ* [70] and intravenously [69], gene therapy with vascular endothelium-derived growth factor (VEGF) [73], and ET [22, 23, 25, 27].

Moderate intensity aerobic ET is responsible for structural and hemodynamic adaptations in the cardiovascular system and promotes adjustments in autonomic nervous system. This can be exemplified by cardiac adaptations such as increase of stroke volume [74, 75], adjustments in diastolic and systolic functions [75], and positive changes in cavities diameter and ventricular mass [76, 77] as well as alterations in the rest heart rate (HR) [77].

Resting bradycardia is associated with a decrease in intrinsic HR as well as an altered autonomic balance, leading to parasympathetic dominance [78]. This is thought to be mediated, at least in part, by an increase in cardiac vagal tone [79]. The mechanisms by which exercise produces changes in autonomic control have yet to be fully understood; however, there is evidence of alterations in the central afferent and efferent pathways and in the effector organs (receptor function) [80, 81].

Aerobic ET is largely recommended for patients with cardiac disease, including those who have experienced an MI. A meta-analysis, based on 48 randomized controlled trials (8940 patients), showed that cardiac rehabilitation programs based on aerobic exercises reduced all-cause mortality by 20% and CVD mortality by 26% in patients after MI, angina pectoris, and/or CAD [14]. Additionally, in another meta-analysis based on 34 studies, the authors observed that aerobic ET (predominantly) after MI, even for a short period (1–3 months), was an effective therapeutic strategy, since the observed benefits extended far beyond the intervention period [16].

One of the most important adaptations to the ET, generally associated with reduced mortality rate, is the improvement in autonomic nervous system regulation. Classic research studies such as Billman et al. [12] and Hull Jr. et al. [13] have suggested that this intervention reduces mortality in individuals after an MI, particularly when associated with increased vagal component and decreased sympathetic activity. La Rovere et al. [82], using head-up tilt testing to evaluate heart rate variability (HRV) in MI patients, have observed that aerobically trained individuals presented a better autonomic response to orthostatic stress, with reduction in vagal modulation and an increase in sympathetic vasoconstrictor outflow due to initiation of the baroreceptors. Moreover, Iellamo et al. [83], in the first randomized controlled study in the area, have observed that aerobic ET results in marked enhancement of both baroreflex sensitivity and HRV in patients with coronary artery disease. These researchers have also suggested that the improvement in baroreflex sensitivity associated with aerobic ET is not limited to patients with prior MI but extends to coronary patients without MI, for whom risk-reducing strategies designed to avoid subsequent lethal events might be of paramount importance.

In la Rovere et al. [7] study, 95 consecutive male patients, survivors of a first uncomplicated MI, underwent four weeks of ET. The researchers found that aerobic ET was associated with increased survival together with an adequate modulation of the autonomic balance toward increase in vagal activity, as revealed by the increase in baroreflex sensitivity. In this context, several clinical studies have reported improvements in cardiovascular autonomic control, particularly in HRV, after ET among MI patients. Sandercock et al. [15] have observed that after eight-week cardiac rehabilitation program, MI participants had significant increases in HRV parameters as compared with those not participating in the training program. In an elegant study, Malfatto et al. [84] have reported improvement in HRV following 8 weeks of aerobic ET. After this period, participants were encouraged to continue exercising at home two to three times per week. After one year of training program participation, improvements in HRV index were still relevant. More recently, six months of aerobic (predominantly) ET significantly decreased muscle sympathetic nerve activity and the low-frequency component of systolic arterial pressure and increased the baroreflex sensitivity in MI patients. These changes were so marked that the differences between patients with MI and the normal control group were no longer observed after ET. These findings highlight the clinical importance of this nonpharmacological therapy based on ET in the long-term treatment of patients with MI [85].

In an experimental setting, our group has studied the role of aerobic ET in functional, biochemical, and molecular alterations, as well as in the mortality rate after MI in rats. Rondon et al. [22] have investigated the role of ET in MI-induced heart failure rats and demonstrated that aerobic ET increased peak oxygen uptake and the high frequency band of HRV, while reducing the low frequency band of HRV. In addition, these authors also observed that after ET protocol the heart failure animals presented improvement in baroreflex control of heart rate and renal sympathetic nerve activity, associated with increased aortic depressor nerve activity. However, we should bear in mind that, although these data suggest an association between increased aortic depressor nerve activity and improvement in baroreflex control, the cause-effect relationship remains unclear. Moreover, Jorge et al. [25] have demonstrated that early aerobic ET intervention (one week) after MI induced an improvement in LV systolic and diastolic functions. In the study, we have also observed a normalization of hemodynamic and regional blood flows and an improvement in cardiovascular autonomic function associated with increased baroreflex sensitivity. These benefits, in turn, resulted in an increase in functional capacity and a reduction in mortality rate in trained infarcted animals. Similar results were also demonstrated in streptozotocin-diabetic rats undergoing MI, for which aerobic ET promoted, in addition to the benefits observed in autonomic function, positive changes in the expression of proteins associated with intracellular calcium handling [86]. Studying female ovariectomized rats undergoing MI, Flores et al. [23] have demonstrated that eight weeks of aerobic ET was able to improve resting hemodynamic status and reflex control of the circulation (arterial and cardiopulmonary baroreflex), possibly associated with the increase in vagal component observed in the study.

In line with la Rovere et al. [7] study, in which aerobic ET was associated with improvement in baroreflex sensitivity and increased survival rate in a 10-year followup, our group tested the hypothesis that autonomic ET benefits might remain for an extended period, even during detraining. Accordingly, in a study by Barboza et al. [27], MI animals underwent three months of aerobic ET (starting one week after MI) and one month of detraining. The authors demonstrated that one month of detraining did not alter the beneficial effects of ET on the MI area, LV morphometry and function, and baroreflex sensitivity, as well as overall survival rate in MI-detrained animals. These findings indicate not only that aerobic ET is an effective tool in the management of cardiovascular and autonomic MI derangements, but also that these positive changes were extended even beyond one month of detraining in rats.

In addition to the benefits of ET on autonomic function, it is now well established that cytokine production by exercise is different from that observed in response to severe infections or tissue injury, since exercises usually do not provide expressive changes in the classic proinflammatory cytokines, TNF-*α* and IL-1*β* [87]. During exercise, IL-6 is the first cytokine present in the circulation, exponentially increasing when the exercise is in course, and fast declining after the exercise period [88–90]. In this sense, there is evidence in the literature that IL-6 may exert anti-inflammatory effects, since circulating IL-6 triggers an inhibitory effect on TNF-*α* and IL-1 production [91] and stimulates the production of IL-1 receptor antagonist and IL-10 [92]. IL-10 and IL-1 receptor antagonist production after exercise, in turn, contributes to the inhibition of the synthesis of a large spectrum of proinflammatory cytokines by different cells, particularly the ones of monocytic lineage [93].

Actually, the protective effects of ET on inflammatory markers have been widely discussed [94]. In this sense, Adamopoulos et al. [95] have demonstrated a reduction in inflammatory markers after 12 weeks of aerobic ET in patients with moderate to severe HF, suggesting a correlation between improvement in exercise tolerance and attenuation ofinflammatory process. In an experimental model of HF, Batista Jr. et al. [96] have shown that aerobic ET in infarcted rats increased the ratio IL-10/tumor necrosis factor-*α* (TNF-*α*) in the soleus muscle of animals, emphasizing the anti-inflammatory effect of exercise after an ischemic event.

Individuals with increased risk of CAD presented reduction in atherogenic cytokines, as interleukin-1 (IL-1), IL-6, TNF-*α*, and C reactive protein, as well as improving atheroprotective cytokines, as IL-10, and transforming growth factor beta-1 after an aerobic ET program [97]. The reduction in plasma TNF-*α*, IL-6, and their receptors has been demonstrated in aerobically trained HF patients [98], suggesting an attenuation in the chronic inflammatory condition mediated by a regulation in peripheral inflammatory response [95, 98]. Similarly, the association between aerobic and resistance ET decreased the concentration of TNF-*α* receptors I and II in patients with HF [99].

Thus, aerobic ET seems to be an important strategy in the management MI-induced changes and HF, especially with regard to improving cardiovascular autonomic control and reducing chronic inflammatory response, consequently reducing cardiac work, decreasing risk of fatal arrhythmias, and increasing survival in affected individuals.

In order to understand the mechanisms associated with autonomic adjustments and anti-inflammatory role of exercise fully, it is necessary to do a more detailed search of the nature, intensity, and duration of ET after MI. The beneficial effects of aerobic ET are well known; however, autonomic and anti-inflammatory effects of resistance ET or high intensity aerobic ET are poorly defined and remain areas for future investigations. In this direction, our group recently demonstrated that resistance ET (moderate intensity) induced additional benefits in the low frequency band (+50%) and high frequency band of HRV (+45%), as well as in the low frequency band of systolic blood pressure variability (−46%) of trained infarcted rats compared to sedentary [100]. These findings suggest that resistance ET was effective in reducing cardiac and peripheral sympathetic modulation, as well as increasing cardiac parasympathetic modulation of infarcted rats.

## 5. Exercise Training on “Inflammatory Reflex” Response after Myocardial Infarction 

Despite all data generated on the anti-inflammatory role of the exercise, the underlying mechanisms associated with improved inflammatory profile in patients after an ischemic event have yet to be fully understood. During the last decade, studies on the anti-inflammatory effects of exercise have pointed to three possible mechanisms [87, 101–106]: reduction in visceral fat mass; increased production and release of anti-inflammatory cytokines from contracting skeletal muscle (myokines); and reduced expression of toll-like receptors (TLRs) on monocytes and macrophages. Regarding the first mechanism, ET is able to reduce low-grade and systemic inflammation via a decrease in proinflammatory adipokine secretion, such as TNF, leptin, retinol-binding protein 4, lipocalin 2, IL-6, and IL-18, which is a direct result of lowering the amount of fat stored in abdominal and visceral depots [107–110].

Related to the second proposed mechanism, during exercise it seems that IL-6, produced by contractile skeletal muscle via a TNF-independent pathway, stimulates systemic appearance of anti-inflammatory cytokines, such as IL-1 receptor antagonist and IL-10, and inhibits the liberation of proinflammatory cytokine TNF-*α* [101, 111]. Additionally, IL-6 may be associated with increase of lipid turnover, stimulating lipolysis as well as fat oxidation [101]. In line with the third proposed mechanism about the anti-inflammatory effects of ET, it has been evidenced that TLRs may be involved in the link between a sedentary lifestyle and inflammation and disease [112]. Studies have observed that blood monocytes from trained individuals present decreased TLR4 expression, which is associated with decreased inflammatory cytokine production, and reduced inflammatory response to endotoxin stimulation* in vitro *[103, 105, 113].

On the other hand, as previously mentioned, the “inflammatory reflex” proposed by Tracey [55] seems to have an important role in the development of the inflammatory process, since it is in line with the suggestion that the activation of the vagus nerve leads to a reduced production of cytokines by the reticuloendothelial system, such as lymph nodes, liver, heart, spleen, and gastrointestinal tract. ET, in turn, modulates cardiac autonomic control with reduction in sympathetic tonus and increases the role of the vagal tonus [7, 15, 85], thereby positively affecting the prognosis of MI patients. Thus, we suggest that in addition to the reduction in visceral fat mass, increased production anti-inflammatory myokines, and reduced expression of TLRs on monocytes and macrophages, the increase in vagal activation induced by ET may be an important mechanism that would explain, at least in part, the improvement in the inflammatory status of animals or patients after MI.

**Figure 3:**
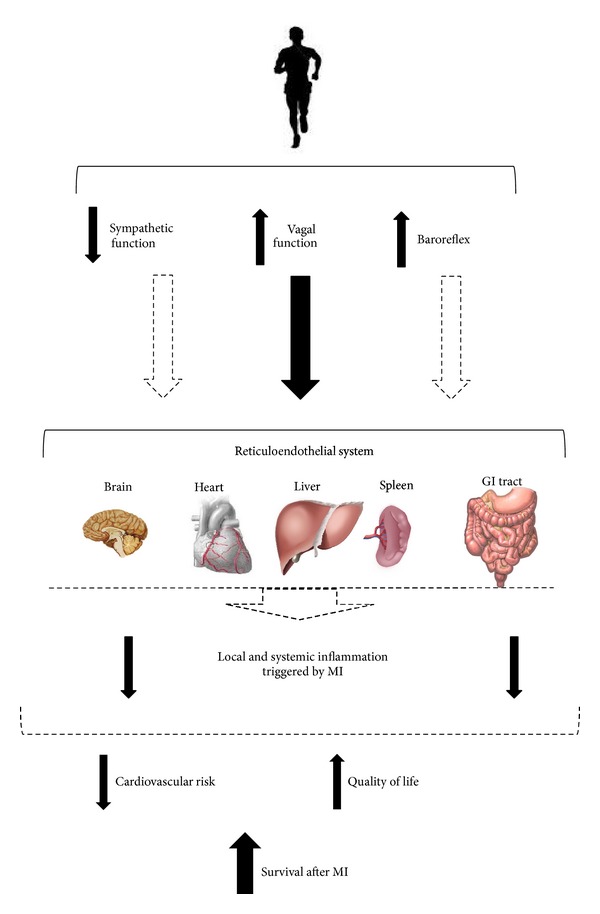
Possible mechanisms associated with inflammatory profile reduction in patients after myocardial infarction undergoing exercise training.

As evidenced in Figure 3, we proposed that chronic benefits of ET would be associated with significant improvements in baroreflex sensitivity, resulting in the increase in vagal activity and reduction in sympathetic activity (as observed to the heart and vessels [7, 12, 13, 15, 22, 23, 25, 27, 78, 82–86]) to important organs (reticulum endothelial system and other tissues), including brain, heart, liver, spleen, and gastrointestinal (GI) tract. This autonomic remodeling, in turn, would be directly associated with improved local and systemic inflammatory profile, reducing cardiovascular risk and enhancing the survival rate in patients who had suffered a MI. When studying infarcted rats undergoing 3 months of moderate-intensity aerobic ET, preliminary data from our group has noted that HRV parameters such as the high frequency band and root mean square of successive difference (RMSSD)—indexes of cardiac parasympathetic modulation—were negatively correlated with cardiac levels of TNF-*α* and IL-6 (unpublished data). Furthermore, the low frequency band of systolic blood pressure variability, an indicator of vascular sympathetic modulation, was positively associated with the TNF-*α* and IL-6 levels in the adipose tissue of these animals (unpublished data). Taken together, our preliminary findings suggest that ET can increase cardiac vagal modulation, as well as reducing sympathetic peripheral modulation, leading to a reduction in the inflammatory profile of infarcted rats.

Despite the vast literature raised in this review, many questions still remain about the benefits of ET after MI on cardiovascular autonomic function and inflammatory response, or if there is a direct relationship between them. At present, we do not know whether there is a cause-effect relationship between the improvement in vagal cholinergic activity promoted by ET and reduction in cardiac and systemic inflammatory status after a cardiac ischemic event. Or even, what is the relative importance of these mechanisms on cardiac morphology and function, as well as in the prognosis of infarcted patients.

Intuitively, we can expect that many anti-inflammatory mechanisms of exercise, known or unknown, are acting in combination in order to better respond to different stimulations applied. Furthermore, it is possible that the involved mechanisms and the magnitude of the benefits found in autonomic function and inflammatory profile will depend on the mode, intensity and duration of exercise performed. However, the vast majority of the studies presented in this review used the moderate intensity aerobic ET. The absence of studies comparing different intensities, durations, and modalities of ET and its possible relationship with the anti-inflammatory cholinergic pathway opens new perspectives for working in exercise cardiology.

## 6. Conclusions

There is consistent experimental and clinical evidence that the autonomic nervous system and inflammatory response play a key role in cardiac and peripheral dysfunctions after myocardial infarction. Exercise training has been associated with increased vagal tonus/modulation, as well as with reduction in sympathetic tonus/modulation and inflammatory profile in heart failure or after myocardial infarction. In recent years, a direct relationship between vagal activity and inflammatory status has been proposed. Here, we suggest that exercise training may improve autonomic function to different sites, leading to a reduction in the proinflammatory response observed after myocardial infarction. However, further experimental and clinical investigations are needed to test the role of exercise training in the modulation of this “inflammatory reflex” after myocardial infarction. The results of these future studies will improve the management of cardiovascular risk, quality of life, and survival of this population.
